# In Situ Generation of Electrolyte inside Pyridine‐Based Covalent Triazine Frameworks for Direct Supercapacitor Integration

**DOI:** 10.1002/cssc.202000518

**Published:** 2020-05-11

**Authors:** Erik Troschke, Desirée Leistenschneider, Tilo Rensch, Sven Grätz, Johannes Maschita, Sebastian Ehrling, Benjamin Klemmed, Bettina V. Lotsch, Alexander Eychmüller, Lars Borchardt, Stefan Kaskel

**Affiliations:** ^1^ Department of Inorganic Chemistry Technische Universität Dresden Bergstraße 66 01069 Dresden Germany; ^2^ Department of Chemical and Materials Engineering University of Alberta 9211-116 Street NW T6G 1H9 Edmonton Alberta Canada; ^3^ Department of Inorganic Chemistry Ruhr-Universität Bochum Universitätsstrasse 150 44801 Bochum Germany; ^4^ Max Planck Institute for Solid State Research Heisenbergstraße 1 70569 Stuttgart Germany; ^5^ Physical Chemistry Technische Universität Dresden Bergstraße 66b 01062 Dresden Germany; ^6^ Ludwig-Maximilians-Universität München (LMU) Butenandtstraße 5-13 (Haus D) 81377 München Germany

**Keywords:** covalent triazine frameworks, cyclotrimerization, nitrogen heterocycles, supercapacitors, waste prevention

## Abstract

The synthesis of porous electrode materials is often linked with the generation of waste that results from extensive purification steps and low mass yield. In contrast to porous carbons, covalent triazine frameworks (CTFs) display modular properties on a molecular basis through appropriate choice of the monomer. Herein, the synthesis of a new pyridine‐based CTF material is showcased. The porosity and nitrogen‐doping are tuned by a careful choice of the reaction temperature. An in‐depth structural characterization by using Ar physisorption, X‐ray photoelectron spectroscopy, and Raman spectroscopy was conducted to give a rational explanation of the material properties. Without any purification, the samples were applied as symmetrical supercapacitors and showed a specific capacitance of 141 F g^−1^. Residual ZnCl_2_, which acted formerly as the porogen, was used directly as the electrolyte salt. Upon the addition of water, ZnCl_2_ was dissolved to form the aqueous electrolyte in situ. Thereby, extensive and time‐consuming washing steps could be circumvented.

## Graphical abstract

**Just add water**: A series of covalent triazine frameworks and derived carbonaceous materials based on 3,5‐dicyanopyridine is developed. These materials, which still contain ZnCl_2_ crucial for the synthesis, are further applied as supercapacitors. The electrolyte is thereby generated in situ by adding water to the electrode set‐up.

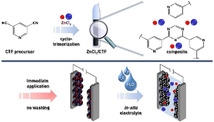

## Introduction

A successful transformation of our current energy supply undoubtedly depends to a great extent on the storage of abundant electrical energy from renewable sources.^1^ To tackle these obstacles in terms of efficient energy storage and management, two complementary technologies, namely, batteries and supercapacitors, have emerged from a myriad of potential systems.^2^ Although battery systems, for example, the conventional lithium‐ion battery, exhibit a high energy density, they suffer from a low power density.^3^, ^4^ Simply put, these trends can be regarded vice versa for supercapacitors because of the dominant physical energy‐storage mechanism.^5^ Supercapacitors (or electrical double‐layer capacitors; EDLCs) usually consist of two oppositely charged electrodes that store energy by the electrosorption of ions at the electrode–electrolyte interface.^4^ The capacitance of these materials relies strongly on a large specific surface area, which is proportional to the achievable capacitance. Fulfilling these requirements, carbon materials have evolved to serve as abundant, cheap, and highly porous electrode materials.^6^ To generate an electrical double layer, a good wettability of the nonpolar carbon electrode with the electrolyte is necessary. Therefore, recent research has focused heavily on the doping of carbon materials with different heteroatoms that can induce local charges on the electrode surface.^7^ Thus, an increase of the surface polarity leads to a better wettability with polar electrolytes.^8^ In contrast to porous carbons, covalent triazine frameworks (CTFs) display adjustable properties, which can be finely tuned by selecting a suitable monomer. In addition, these porous polymers provide full control over the degree of N doping and, more importantly, over the incorporated N species.^9^ The CTFs were synthesized by a trimerization reaction of aromatic nitriles under ionothermal conditions in molten ZnCl_2_.^10^

Here, ZnCl_2_ serves multiple purposes, namely, as the solvent, Lewis acid to trigger the trimerization, and porogen. After the synthesis, which is usually performed in an ampoule, the pristine material is obtained but still contains a large amount of ZnCl_2_, which is dispersed finely throughout the whole sample. Hence, an extensive washing procedure is required to obtain the CTF material, which can be consequently applied as electrode material in a supercapacitor. Ironically, an electrolyte, which consists of a dissolved salt, must be added before the evaluation of the electrochemical performance. Bypassing multiple washing steps would drastically facilitate the efforts towards the final application and would save huge amounts of solvents. In the course of the synthesis, ZnCl_2_ is located within the pores of the CTF material. Hence, the addition of water should generate an aqueous ZnCl_2_ electrolyte in situ and simultaneously unblock the pores of the CTF. This method, namely, the “in situ electrolyte concept”, has been applied recently for K_2_CO_3_‐activated carbons to form aqueous electrolytes.^11^ Electrolytes based on water‐soluble salts such as ZnCl_2_ have not been investigated widely as an electrolyte system in supercapacitors yet. However, it certainly fulfills the prerequisites of an aqueous electrolyte such as ionic conductivity and good solubility, such as conventional Li_2_SO_4_‐ or H_2_SO_4_‐based electrolytes.^2^ CTFs have been designed from various monomeric building blocks that include heterocyclic polycarbonitriles.^12^ Monomers such as pyridine,^13^ carbazole, or furan derivatives^14^ enable the valuable introduction of exposed heteroatoms to adjust the adsorption properties or to establish coordination sites precisely. A remarkable example is the development of a heterogeneous Periana catalyst to convert methane to methanol based on a 2,6‐dicyanopyridine CTF.^15^ Indeed, they have been applied in supercapacitor applications,^16^ but a common drawback of CTFs is a lack of conductivity because they are known to be semiconducting at standard reaction temperature, that is, 400 °C.^17^ To overcome this particular issue, higher reaction temperatures are required to induce conductivity.^18^ Elevated reaction temperatures are further known to be accompanied by a huge increase of porosity.^19^ In parallel, the well‐defined porous polymer is partially decomposed because of carbonization processes that lead to an N‐doped carbon rather than a polymer.

**Figure 1:**
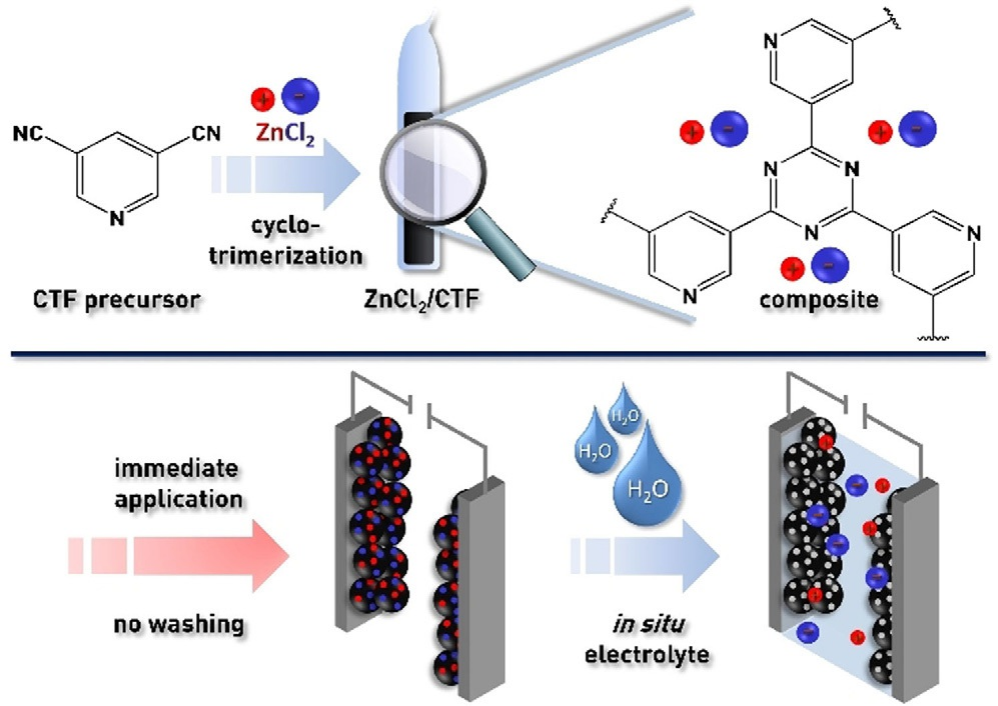
Synthesis of the 3,5‐dicyanopyridine CTF and immediate application as a supercapacitor without further purification.

In this contribution, a series of new CTFs and derived carbonaceous materials based on 3,5‐dicyanopyridine is established. By exploiting the precisely adjustable properties of the materials, for the first time, a straightforward approach for CTF compounds is showcased that avoids extensive washing by using the porogen (ZnCl_2_) as an electrolyte salt directly and, consequently, allows immediate application as electrode material in a supercapacitor (Figure 1).

## Results and Discussion

### Material synthesis and characterization

We developed our new CTF with a 3,5‐dicyanopyridine (DCP) monomer. In principle, this monomer exhibits a high degree of pyridinic N atoms and the ability to generate highly porous frameworks. A striking difference is displayed by the reduced steric hindrance of the 3,5‐substituted monomer in contrast to well‐known 2,6‐dicyanopyridine (2,6‐DCP).^20^ Thus, the formation of highly porous open frameworks that bear exposed pyridinic N atoms should be favored by the use of DCP in contrast to the use of 2,6‐DCP, which is known to lead to dense ZnCl_2_‐monomer assemblies and to networks with moderate porosity.^20^

The new DCP‐CTF was synthesized under ionothermal conditions in molten ZnCl_2_. Four different materials were obtained by applying synthesis temperatures of 400, 500, 600, and 700 °C. Consequently, a large impact on the porosity was anticipated because of a thermally induced structural evolution. Before the analysis of the structural properties, the samples were purified to remove residual ZnCl_2_ (henceforth, referred to as DCP‐CTF‐*X*; in which *X* is the synthesis temperature).

**Figure 2:**
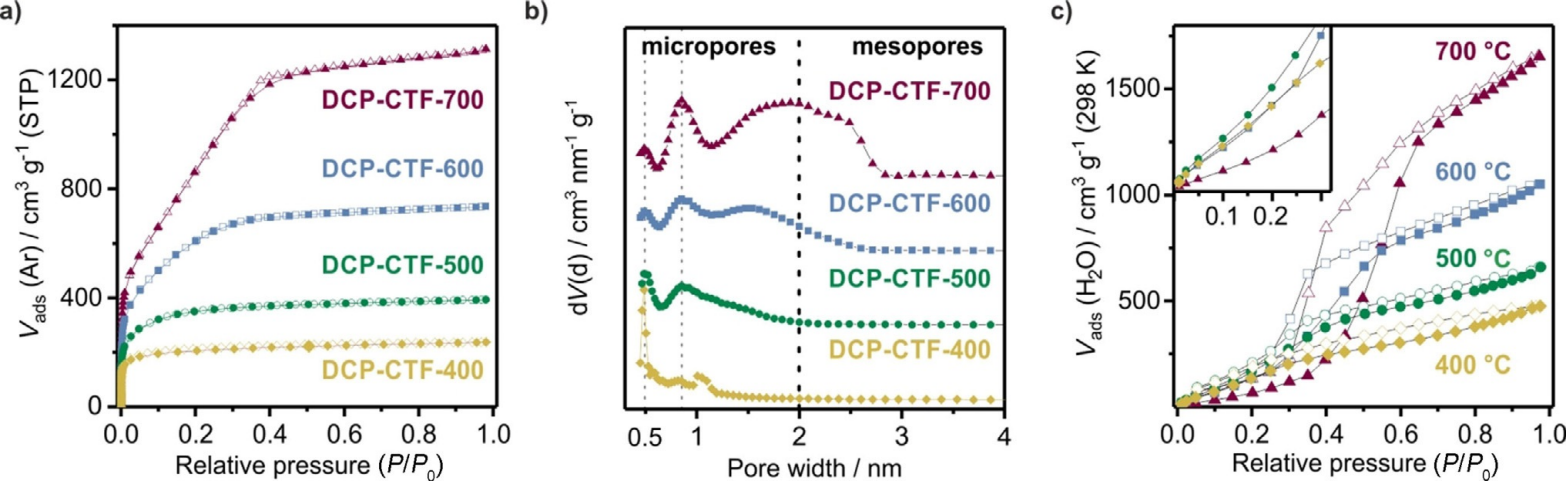
a) Ar physisorption (87 K) isotherms that reveal a temperature‐induced evolution of porosity. b) Pore‐size distribution (QSDFT). c) Water physisorption isotherms (298 K).

To investigate the porosity of these N‐rich porous polymers, we performed Ar physisorption at 87 K (Figure 2 a). As a result of its indifference to the polar surfaces of the materials, Ar as an adsorptive is more convenient than N_2_ to determine surface areas and especially pore size distributions accurately.^21^ All isotherms reveal a steep increase in the low‐pressure regime, which indicates pronounced microporosity. Furthermore, the isotherms can be classified as type I(a) for the samples synthesized at 400 (DCP‐CTF‐400) and 500 °C (DCP‐CTF‐500) and type I(b) for the samples generated at higher temperatures (Figure S4 in the Supporting Information). This description for DCP‐CTF‐600 and DCP‐CTF‐700 reflects a continuous uptake of Ar until a relative pressure (*P*/*P*_0_) of 0.4, which can be interpreted as a result of the presence of smaller mesopores. To estimate the specific surface area, the Brunauer–Emmett–Teller (BET) model was applied. Based on the adsorption experiments, BET surface areas that cover a broad range from 680 (for DCP‐CTF‐400) to 3120 m^2^ g^−1^ (for DCP‐CTF‐700) could be achieved (see the Supporting Information for all physisorption data). Thus, the thermal treatment of the samples yielded highly porous N‐rich samples that should lose their polymeric character with the increase of temperature. The quenched solid density functional theory (QSDFT) method developed for carbons (slit pores, equilibrium kernel) was applied to determine the pore‐size distributions (Figure 2 b). Clearly, all materials show a narrow pore‐size distribution. As mentioned above, DCP‐CTF‐400 is a purely microporous material, which was confirmed by the presence of ultra‐micropores with a diameter of 0.5 nm. All samples obtained at higher reaction temperatures display super‐micropores of 0.8 nm and an enhanced degree of smaller mesopores with a maximum diameter of 3 nm (at 600 and 700 °C). In addition to Ar physisorption, low‐pressure CO_2_ adsorption at 273 K (1 bar) was conducted (Figure S5 in the Supporting Information) and revealed an extremely high CO_2_ adsorption capacity of 6.6 mmol g^−1^ for DCP‐CTF‐600. This value is among the highest ever reported for CTFs and related porous organic polymers [hexaazatriphenylene‐based CTF (HAT‐CTF): 6.3 mmol g^−1^;^22^ bipyridine‐based CTF (bipy‐CTF600): 5.6 mmol g^−1^].^23^ Water‐adsorption experiments were performed to estimate the affinity towards water, which might be enhanced because of a high degree of N‐doping (Figure 2 c). For DCP‐CTF‐600 and DCP‐CTF‐700, assumed to be the most hydrophobic of this series, type V isotherms were observed. Thus, weak adsorbent–adsorbate interactions are indicated by a low uptake at low relative pressures (*P*/*P*_0_=0.0–0.4). In contrast, DCP‐CTF‐400 and DCP‐CTF‐500 do not exhibit a pronounced uptake at high relative pressures. This might be because of the absence of mesoporosity and/or a more hydrophilic character. Generally, the adsorbed amount of water at full saturation (*P*/*P*_0_=0.98) increases with the reaction temperature to reach a huge capacity of 1650 cm^3^ g^−1^ for DCP‐CTF‐700. Clearly, this trend follows that of Ar physisorption and can be attributed to the increasing pore volume within this series of samples.

**Figure 3:**
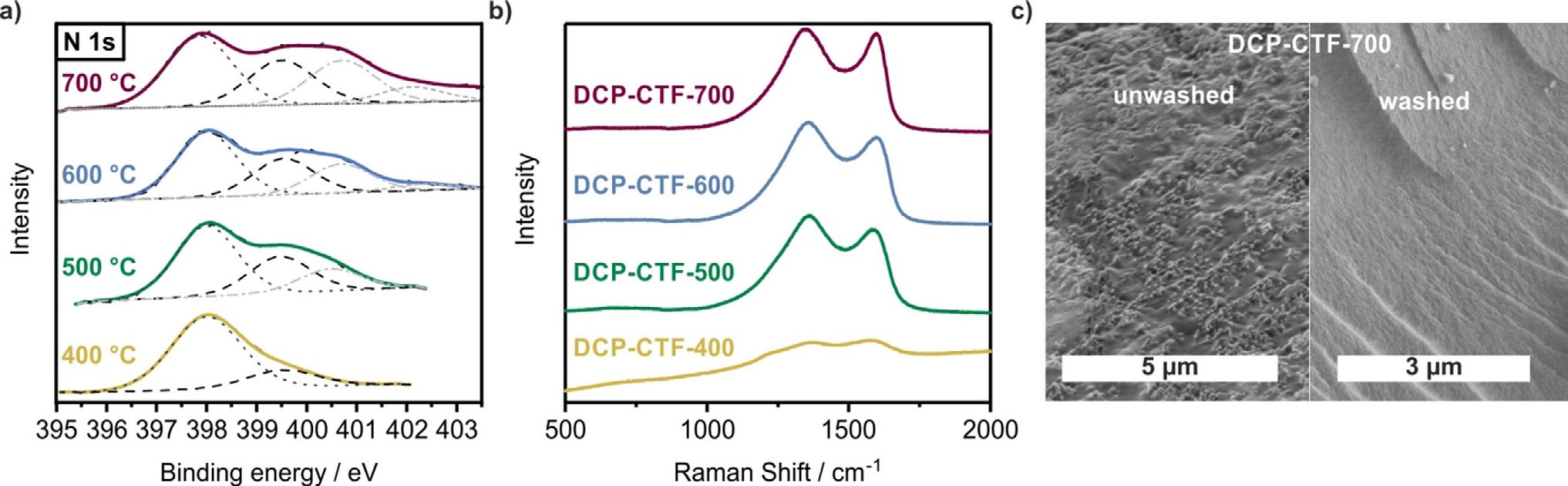
a) Deconvoluted N 1s XPS spectra that show pyridinic (BE≈398.0 eV), pyrrolic (BE≈399.5 eV), quaternary (BE ≈400.7 eV), and oxidized N species (BE≈402.3 eV). b) Raman spectra of DCP‐CTF samples that reveal a different degree of carbonization. c) SEM pictures of DCP‐CTF‐700 that compare an unwashed and washed sample.

To further estimate the different N contents within this set of samples, elemental analysis (EA) was conducted. As a result of the presence of adsorbed water under ambient conditions, careful activation was necessary before elemental analysis was performed. Close to ideal values for the C/H (C/H_ideal_=2.3) and C/N (C/N_ideal_=2.3) ratios, which are well suited to evaluate the degree of carbonization, were determined for DCP‐CTF‐400 (C/H=2.0; C/N=2.5). As expected, at elevated reaction temperatures, an increase of the C/H and C/N ratios is detected (Table S1 in the Supporting Information). Thus, CTF‐DCP‐700 shows a C/H ratio of 8.5 and a C/N ratio of 6.9. From these findings, the expected trends of an increased degree of carbonization accompanied by a loss of N could be confirmed. Because a structural rearrangement as a result of elevated reaction temperatures is expected, X‐ray photoelectron spectroscopy (XPS) was performed to monitor the transition (Figure 3 a). As a result of the broad range of N 1s binding energies, XPS is a powerful tool for the characterization of CTFs and to distinguish between different N species. If we consider an ideal polymerization, the material should contain pyridinic (or triazinic) aromatic N with a binding energy (BE) of approximately 398.0 eV exclusively.^24^ Deconvolution of the spectrum of DCP‐CTF‐400 shows a broad peak at BE=398.0 eV, which represents a well‐retained structure. A shoulder of the peak at a higher BE (≈399.5 eV) may be induced by pyrrolic N species. Additionally, metal coordination should be considered but is less probable because no Zn is detected in the survey XPS spectrum of DCP‐CTF‐400 (Figure S7 in the Supporting Information).^24^ This observation can be rationalized by a structural reorganization of the scaffold, either caused by irreversible C−C or C−N bond formation under Lewis acidic conditions (e.g., cycloaddition reactions) or carbonization of the material. For the samples synthesized at higher reaction temperatures, the second peak (“decomposition peak”) at higher BEs becomes even more pronounced (see Table S3 in the Supporting Information for peak areas and ratios). Moreover, deconvolution of the N 1s XPS spectra of DCP‐CTF‐500, DCP‐CTF‐600, and DCP‐CTF‐700 reveals a third peak with a higher BE of approximately 400.7 eV, which can be assigned to quaternary N atoms.^24^ Because an ongoing carbonization is expected, the formation of graphitic layers takes place, and, consequently, quaternary N species are introduced into these layers. This effect is reflected in the increase of the peak area of the quaternary N species up to 700 °C (DCP‐CTF‐700), which supports the impact of the reaction temperature on the structure strongly. A structural change is also indicated by the C 1s spectra (Figure S8 in the Supporting Information). As expected, for DCP‐CTF‐400 exclusively C−C sp^2^ C (BE≈284.2 eV), C=N (BE≈285.5 eV, C in the triazine core), and oxidized C species (C−O, BE≈286.3 and 288.2 eV) can be detected.^24^, ^25^ In contrast, C−C sp^3^ C (BE≈284.7±0.3 eV) is observed in the spectra of the high‐temperature samples. Interestingly, in the spectrum of DCP‐CTF‐600, the peak area for sp^3^ C (and also the sp^3^/sp^2^ ratio) reaches a maximum of 40 % and decreases again in the spectrum of DCP‐CTF‐700 (peak area=28 %). Thus, DCP‐CTF‐600 can be considered as a structural transition between the polymer and a graphitized structure.

Raman spectroscopy was conducted to further evaluate the structural changes of the polymeric system upon thermal treatment (Figure 3 b). As expected, for porous carbon materials, the typical appearance of a d band at approximately $\tilde{\nu }$ =1350 cm^−1^ and a G band at approximately $\tilde{\nu }$ =1600 cm^−1^ is observed. The d band is caused by the breathing mode of sp^2^ C atoms in aromatic rings and becomes active in the presence of defects. In contrast, the G band includes the in‐plane bond‐stretching motion of sp^2^ C atoms in graphitic carbon parts and does not require the presence of aromatic rings.^26^, ^27^

In the spectrum of DCP‐CTF‐400 almost no intensity of the aforementioned vibrations is detectable. This might be because of the polymeric character of the sample. Thus, strong background fluorescence is present for this sample, and no pronounced vibrations are visible.^28^ Contrary to these findings, for all samples obtained at elevated reaction temperatures, an evolution of Raman activity and, consequently, the presence of the D and G bands is determined. With regard to the peak intensity ratio *I*(D)/*I*(G) of the full width at half maximum, a decrease of the value with an increase of the reaction temperature (500 °C: I(*D*)/I(*G*)=3.88 to 700 °C: I(*D*)/I(*G*)=2.60) is determined (Table S4 in the Supporting Information). This trend might be caused by different effects in parallel. With an increase of the temperature, an increase of the degree of disorder is anticipated. Simultaneously, as a consequence of the reorganization of the polymeric scaffold towards a carbonaceous material, polymeric ring structures will break, and the number of C atoms that contribute to the intensity of the G band will increase. Both concomitant effects can be interpreted as a significant reorganization within the initially polymeric system towards a carbon‐like material.

The powder (PXRD) patterns demonstrate that all samples are amorphous (Figure S9 in the Supporting Information). A certain degree of long‐range order, however, is an only rarely observed feature for CTFs.^10^, ^29^, ^30^ We performed thermogravimetric analysis (TGA) under synthetic air to reveal a high thermal stability up to 400 °C for all materials. Similar trends for the stability were found for TGA experiments performed under Ar (see the Supporting Information for TGA data). We used SEM to reveal a rough surface texture with residual small crystallites of the reaction medium ZnCl_2_. If we compare an unwashed sample with the washed analogue, a clear difference can be observed (Figure 3 c). Before washing, ZnCl_2_ covers the rough surface homogeneously. After washing, the surface texture changes significantly and appears to be smooth with a few cavities. However, the porosity of the materials cannot be judged by using SEM.

### Direct application as a supercapacitor using the porogen as an electrolyte

The CTF/ZnCl_2_ composites were applied and characterized as symmetric EDLCs without any further purification step. Therefore, the materials were processed directly into electrodes after ionothermal synthesis (referred to as CTF/ZnCl_2_‐*X*, in which *X* is the synthesis temperature). Based on the amount of starting material, the electrode contains an excess of ZnCl_2_ (84 wt %). By adding purified water, the electrolyte is generated in situ in the porous framework (16 wt %). To this end, water (0.1 mL) was added to an electrode mass of 48 mg, which leads to an electrolyte concentration of 403 g L^−1^ of salt in water. If we assume that the electrolyte salt consists purely of residual ZnCl_2_, the molar concentration is calculated to be 2.96 mol L^−1^.

**Figure 4:**
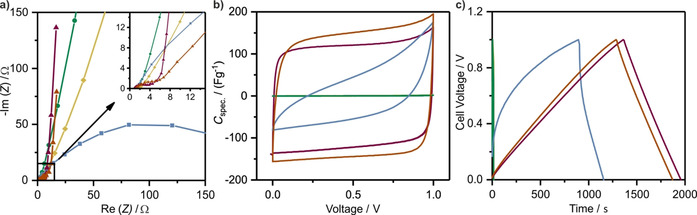
a) Nyquist plot; b) CVs at 10 mV s^−1^; c) Galvanostatic charge–discharge cycles at 1 A g^−1^. Sample code: CTF/ZnCl_2_‐400 (yellow); CTF/ZnCl_2_‐500 (green); CTF/ZnCl_2_‐600 (blue); CTF/ZnCl_2_‐700 (red); DCP‐CTF‐700 (orange).

With regard to the Nyquist plot (Figure 3 a), the capacitive behavior depends on the synthesis temperature. From 400 to 500 °C the energy‐storage mechanism becomes more capacitive as reflected by a sharper increase at lower frequencies. An increase of the temperature to 600 °C leads to a dramatic increase of the resistance of the system. This trend is confirmed by the large equivalent distributed resistance (EDR). We anticipate the polymeric framework to be partially converted into a carbonaceous material at higher temperatures, which would result in a higher resistance and an inferior capacitive performance. It is assumed that the material synthesized at 600 °C can be interpreted as a transition state between a semiconductor and a conductor. This observation might be explained by a stepwise change of the mechanisms responsible for conductivity. Although the CTF material at 400 °C exhibits conductivity based on its conjugated π‐system and potential charge‐transfer processes that stem from N functionalities, the material obtained above 600 °C shows graphitic domains (Figure 3 b). These domains provoke an enhanced conductivity as present in carbon materials, which induces the EDR decrease observed for CTF/ZnCl_2_‐700. The conductivity loss of CTF/ZnCl_2_‐600 correlates with the transition between these two conductivity mechanisms. This hypothesis of carbonization–decomposition is supported strongly by the results obtained by using XPS and EA. Moreover, the cyclic voltammograms (CV) and the galvanostatic charge–discharge curves demonstrate the transition from noncapacitive to capacitive energy storage (Figure 4 b, c). CTF/ZnCl_2_‐400 and CTF/ZnCl_2_‐500 do not show any capacitance (Table 1). We attribute these findings to the polymeric character of the electrodes, which leads to stronger interactions between the electrode surface and the electrolyte ions in contrast to a pure electrosorption. Therefore, CTF/ZnCl_2_‐400 and CTF/ZnCl_2_‐500 do not act as EDLCs, and a capacitance cannot be developed.

**Table 1:** Electrochemical data.

Sample	Electrolyte	*C*_Spec._^[a]^ [F g^−1^]	*E*_Spec._^[b]^ [W h kg^−1^]	EE^[c]^ [%]
CTF/ZnCl2‐400	in situ	0	0	0
CTF/ZnCl2‐500	in situ	0	0	0
CTF/ZnCl2‐600	in situ	60	2.45	59
CTF/ZnCl_2_‐700	in situ	141	3.79	69
DCP‐CTF‐700	2.96 m ZnCl_2_	154	3.76	69
DCP‐CTF‐700	1 m Li_2_SO_4_	129	3.79	78

[a] Calculated with discharge at 1 A g^−1^, normalized to the carbon mass in one electrode. [b] Specific energy, calculated with discharge at 1 A g^−1^. [c] Energy efficiency, calculated as a quotient of the specific energy obtained from discharge and charge at 1 A g^−1^.

A structural change at 600 °C is indicated by a nonrectangular CV shape and a curved discharge graph. This finding implies a transition state between a polymeric and a carbonaceous scaffold. Hence, for CTF/ZnCl_2_‐600, a specific capacitance of 60 F g^−1^ was calculated from the discharge cycle. In contrast, the specific capacitance of the supercapacitor derived from unwashed CTF/ZnCl_2_‐700 (Table 1) is 141 F g^−1^. Moreover, CTF/ZnCl_2_‐700 shows a typical rectangular CV shape as expected for a porous supercapacitor material that electrosorbs electrolyte ions on its surface. This further supports the assumption that it is a purely carbon‐like porous network.

After we evaluated the electrochemical performance of the unwashed CTF samples by taking advantage of an in situ generated electrolyte, we compared these results to supercapacitors derived from the purified CTF samples. To maintain a comparable electrolyte concentration, a 2.96 m ZnCl_2_ solution (equal to the calculated in situ electrolyte concentration) was used as the electrolyte. The similarity of both measurement procedures is reflected in the comparable CVs and galvanostatic charge–discharge cycles obtained (Figure 4 b, c). In addition to similar curve shapes, the specific capacitances are in the same range: 141 F g^−1^ for the nonpurified and 154 F g^−1^ for the purified sample (Table 1). A difference of merely 13 F g^−1^ can be ascribed to miniscule weighing errors or an incomplete dissolution of the electrolyte salt in the in situ supercapacitor. However, as demonstrated by these results and the almost identical specific energies of 3.79 Wh kg^−1^ for the in situ generated electrolyte and 3.76 Wh kg^−1^ for the ZnCl_2_ reference electrolyte, the new approach enables the direct integration of a nonpurified CTF material into a supercapacitor. Moreover, an even distribution of the salt and accessibility of all pores is guaranteed without restrictions that arise from wetting problems. Even without purification of the material in advance, this method allows an estimation of the material properties based on their behavior as a supercapacitor. We can turn the drawback of purification into a benefit by using ZnCl_2_ as an inexpensive electrolyte precursor, and this concept is promising to be time‐efficient and environmentally benign.

## Conclusions

We presented the synthesis of a new pyridine‐based covalent triazine framework (CTF) and its straightforward application as supercapacitor by the utilization of residual ZnCl_2_ as a precursor for an in situ generated electrolyte. By synthesizing a set of polymeric and carbonaceous materials based on the pyridine CTF, the material properties were showcased to be finely adjustable as demonstrated by surface areas that range from 680 to 3120 m^2^ g^−1^. The results of CO_2_ adsorption also revealed a potential future application as an efficient CO_2_‐capture material. Moreover, a detailed X‐ray photoelectron spectroscopy (XPS) study was conducted to monitor the transition of the polymer into a carbonaceous material upon thermal treatment. From these structural insights, a straightforward concept is demonstrated that bypasses extensive washing of the CTF materials and allows the immediate application of the nonpurified CTF/ZnCl_2_ composites as electrode materials in a supercapacitor with the aid of an in situ generated electrolyte. Importantly, the temperature‐dependent and structure‐related electrochemical performance of the different CTF materials was fully correlated with the structural indications obtained by using XPS. Thus, the in situ generation of electrolyte is proven to be environmentally benign and, most decisively, it is shown to provide insights into structural features of the investigated CTF material without the accumulation of waste caused by extensive washing.

## Experimental Section

### Materials and reagents

Zinc chloride (ABCR, anhydrous, 98 %) was stored in a glovebox and used as received. 3,5‐Dicyanopyridine was synthesized according to the method of Šturala et al.^31^

### General methods

Elemental analyses were performed by using a vario MICRO cube Elemental Analyzer from Elementar Analysatorsysteme GmbH in CHNS modus. The samples were activated at 200 °C before the measurement.

Ar physisorption measurements were performed at 87 K by using an Autosorb‐IQ‐C‐XR (Quantachrome Instruments) with high‐purity gas (Ar: 99.999 %). Specific surface areas (*S*_BET_) were calculated by using the BET equation in a relative pressure range that fits the consistency criteria proposed by Rouquerol et al.^32^ Pore‐size distributions were calculated by using the QSDFT method for carbon (slit pores, equilibrium kernel) using the adsorption branch. Ultra‐micropore volumes were calculated from the cumulative pore volumes at a pore diameter of less than or equal to 0.7 nm. Super‐micropore volumes were determined by subtracting the respective ultra‐micropore volume from the cumulative pore volume at a pore diameter of 2 nm (or micropore volume). Mesopore volumes were determined by subtracting the respective micropore volume from the cumulative pore volume at a pore diameter of 26 nm. Total pore volumes were measured at *P*/*P*_0_=0.95. Before physisorption experiments, all samples were activated at 503 K for 24 h under vacuum.

CO_2_ physisorption measurements were performed at 273 K by using an Autosorb 1C (Quantachrome Instruments, USA) with high‐purity gas (CO_2_: 99.995 %, Air Liquide).

PXRD patterns were collected in transmission geometry (MYTHEN 1 K detector) by using a STOE STADI P diffractometer operated at 40 kV and 30 mA with a Ge monochromator using CuK_α1_ radiation.

XPS measurements were conducted by using a Theta Probe photoelectron spectrometer (Thermo Scientific). The spectrometer was equipped with a monochromatic AlK_α_ X‐ray source of 100 W at 15 kV. The kinetic energy of the photoelectrons was determined with the hemispheric analyzer set to pass energy of 200 eV for wide‐scan spectra and 50 eV for high‐resolution spectra. Later, all recorded peaks were shifted by the same value that was necessary to set the C 1s sp^2^ component peak to BE=284.20 eV. Quantitative elemental compositions were determined from the integrals of the Gauss–Lorentz fit functions. High‐resolution spectra were deconvoluted by using the Casa XPS deconvolution software. A linear spectrum background was subtracted. The deconvolution of spectra was performed using a mixed Gauss–Lorentz function in which the Lorentzian contribution was set to 30 % and a full width at half maximum that was kept constant within one sample. Free parameters of component peaks were their BE and height.

Raman spectroscopy was performed by using a RM‐2000 from Renishaw with a 50× objective (NA=0.75) and a wavelength of 532 nm.

SEM images were obtained by using a Hitachi SU8020 SEM equipped with a secondary electron (SE) detector. Before the measurement, the samples were prepared on an adhesive carbon pad and sputtered with gold to obtain the necessary electron conductivity.

Water physisorption measurements were performed at 298 K by using an Autosorb‐IQ‐C‐XR (Quantachrome Instruments).

TGA was performed with a ramp rate of 5 K min^−1^ in either a flow of synthetic air or Ar by using a Netzsch STA 409 PC Luxx.

A Nabertherm P330 oven was used as a heating device for all syntheses.

### General synthesis procedure for DCP‐CTFs

CTF syntheses were performed in ampoules prepared under inert atmosphere (in a glovebox). Typically, 3,5‐dicyanopyridine (0.50 g, 3.9 mmol, 1 equiv.) and ZnCl_2_ (2.64 g, 19.5 mmol, 5 equiv.) were mixed by grinding and transferred into quartz ampoules. The ampoules were sealed under vacuum and heated (for temperature program see the Supporting Information). The ampoules were then cooled to RT and opened. The reaction product was ground and then stirred in 1 n HCl for 24 h at 60 °C. The sample was then washed thoroughly with water, *N*,*N*‐dimethylformamide, and acetone to remove residual salt and organic impurities. After washing, the resulting black powder was dried at 150 °C.

### Electrode preparation and electrochemical characterization

The CTF composite materials were prepared as free‐standing electrodes. They were dispersed in ethanol, and 5 wt % polytetrafluoroethylene (PTFE, granular) as a binder was added. By crushing the mixture in an agate mortar, a dough‐like mass was obtained, which was further rolled until the electrodes had a thickness of approximately 150 μm. The electrodes were dried in a vacuum oven at 120 °C for 24 h, and a disc cutter was used to obtain electrodes with a diameter of 10 mm. The measurements were conducted in custom‐built cells. A 12 mm diameter Whatmann GF/D was used as a separator.

The electrochemical measurements were performed by using a Biologic VMP3 potentiostat/galvanostat. The specific capacitances were calculated according to Equation (1) from the galvanostatic discharge curves at with a specific current of 1 A g^−1^.

(1) $$C_{spec.}=\;\frac{4\;Q}{U_{corr}\times m\;}$$

in which *Q* is the charging of the discharge cycle, *U*_corr_ is the *iR*‐drop‐corrected cell voltage, and *m* is the mass of CTF in both electrodes.

Potential limited electrochemical impedance spectroscopy (PEIS) was performed in a frequency range of 100 kHz to 10 mHz with an amplitude of 10 mV.

CV was performed from 0 to 1 V with different scan rates from 5 to 500 mV s^−1^.

## Conflict of interest

*The authors declare no conflict of interest*.

## Supporting information

As a service to our authors and readers, this journal provides supporting information supplied by the authors. Such materials are peer reviewed and may be re‐organized for online delivery, but are not copy‐edited or typeset. Technical support issues arising from supporting information (other than missing files) should be addressed to the authors.

Supplementary
