# Supplementary material for: In Situ Generation of Electrolyte inside Pyridine‐Based Covalent Triazine Frameworks for Direct Supercapacitor Integration
Source: ChemSusChem. 2020 May 11;13(12):3192–8. doi: 10.1002/cssc.202000518 (PMC7317966; doi:10.1002/cssc.202000518)
Supplement: Supplementary file 1 — Supplementary [file CSSC-13-3192-s001.pdf]

ChemSusChem

Supporting Information

In Situ Generation of Electrolyte inside Pyridine-Based Covalent Triazine Frameworks for Direct Supercapacitor Integration

Erik Troschke+,[a] Desirée Leistenschneider+,[b] Tilo Rensch,[c] Sven Grätz,[c] Johannes Maschita,[d, f] Sebastian Ehrling,[a] Benjamin Klemmed,[e] Bettina V. Lotsch,[d, f] Alexander Eychmüller,[e] Lars Borchardt,*[c] and Stefan Kaskel*[a]

Table of contents

1. Monomer synthesis 2
2. CTF Synthesis 5
3. Elemental analysis (EA) 5
4. Nitrogen physisorption (77 K) 6
5. Pore Size Distribution (N_2) 8
6. Carbon dioxide physisorption (273 K) 9
7. X-ray photoelectron spectroscopy 10
8. Infrared spectroscopy (IR) 12
9. Powder X-ray diffraction (PXRD) 14
10. Thermogravimetric analysis 15
11. Scanning electron microscopy (SEM) 16
12. Electrochemical characterization 17

References 19

1. Monomer synthesis

In general, all organic syntheses were performed under argon athmosphere. The syntheses were conducted using slight modifications of the protocol of Šturala et al..[1]

Synthesis of Pyridine-3,5-dicarboxamide

Pyridine-3,5-dicarboxylic acid (19.5 g, 0.12 mol, 1 eq.) was suspended in dry dichloromethane (250 mL). Freshly distilled thionyl chloride (60 mL, 0.83 mol, 7 eq.) and dry N,N-dimethylformamide (4 mL) were added and the resulting mixture was heated to reflux until the acid was completely dissolved. Then, the reaction mixture was heated over night. The solvent and the excess of thionyl chloride were removed in vacuo. The resulting acyl chloride was dissolved in benzene (150 mL) and evaporated under reduced pressure. The residual solid was dissolved in dioxane (500 mL) and aqueous ammonia (600 mL) was added. After 60 min, the white precipitate was collected by filtration, washed with water (8 × 100 mL) and dried in vacuo to give pyridine-3,5-dicarboxamide (11.85 g, 0.072 mol, 62 %) as a white solid.

^{1}H-NMR (DMSO-d$_6$): δ [ppm]: = 9.12 (d, J = 2.16 Hz, 2 x CH_{arom}), 8.63 (t, J = 2.16 Hz, 1 x CH_{arom}), 8.25 (br s, 2 x NH), 7.69 (br s, 2 x NH).

^{13}C-NMR (DMSO-d$_6$): δ [ppm]: = 166 (2 x $CONH_2$), 150 (2 x CN_{arom}), 134 (2 x $C_{q,arom}$), 129 (1 x CH_{arom}).

FT-IR: v [cm^{-1}] = 3400 (v_{N-H}), 3320 (v_{C-H}), 3160 (v_{C-H}), 3100 (v_{N-H}), 1690 (amide I), 1620 (amide II), 1580 ($v_{C=C}$), 1460 ($v_{C=C}$), 1400 ($v_{C=C}$), 1390 (v_{C-N}), 1240 (amide III), 1120 ($\delta_{C=C\ ip}$), 1100 ($\delta_{C=C\ ip}$), 1020 ($\delta_{C=C\ ip}$), 800 ($\delta_{ringbending}$), 730 ($\delta_{C-H\ deformation\ oop}$), 690 ($\gamma_{C-H}$), 680 ($\delta_{O=C-NH2\ oop}$).

EA: calculated: C: 50,91 % H: 4,27 % N: 25,44 % O: 19,38 % C/H: 1,00 C/N: 2,33
found: C: 49,81 % H: 4,17 % N: 24,14 % C/H: 1,00 C/N: 2,41

Synthesis of 3,5-dicyanopyridine

Pyridine-3,5-dicarboxamide (10.3 g, 62.4 mmol, 1 eq.) was suspended in dry N,N-dimethylformamide (250 mL). Freshly distilled phosphoryl trichloride (34 mL, 0.37 mol, 5.8 eq.) was added dropwise and a clear orange solution was resulting. The mixture was stirred for 3.5 h at room temperature and allowed to stand overnight. The excess of phosphoryl trichloride was quenched and neutralized with aqueous $NaHCO_3$. The mixture was extracted with ethyl acetate (5 × 300 mL). The organic layers were combined, dried with magnesium sulfate, filtrated and evaporated in vacuo. The crude product was purified by sublimation (70 °C, 10^{-3} mbar) to give 3,5-dicyanopyridine (6.9 g, 53 mmol, 85.3 %) as a white crystalline solid.

^{1}H-NMR ($CDCl_3$-d$_1$): δ [ppm]: = 9.12 (d, J = 2.07 Hz, 2 x CH_{arom}), 8.25 (t, J = 2.07 Hz, 1 x CH_{arom}).

^{13}C-NMR ($CDCl_3$-d$_1$): δ [ppm]: = 155 (2 x CN_{Nitril}), 141 (2 x CN_{arom}), 113 (2 x $C_{q,arom}$), 110 (1 x CH_{arom}).

FT-IR: v [cm^{-1}] = 3040 (V_{C-H}), 3020 (V_{C-H}), 3000 (V_{C-H}), 2900 (V_{C-H}), 2240 ($V_{nitrile}$), 1560 ($V_{C=C}$), 1410 ($V_{C=C}$), 1130 ($\delta_{C=C\ ip}$), 920 ($\delta_{ringbending}$), 700 (γ_{C-H}).

EA: calculated: C: 65.11 % H: 2.34 % N: 32.54 % C/H: 2.33 C/N: 2.33
found: C: 65.03 % H: 2.18 % N: 32.76 % C/H: 2.49 C/N: 2.32

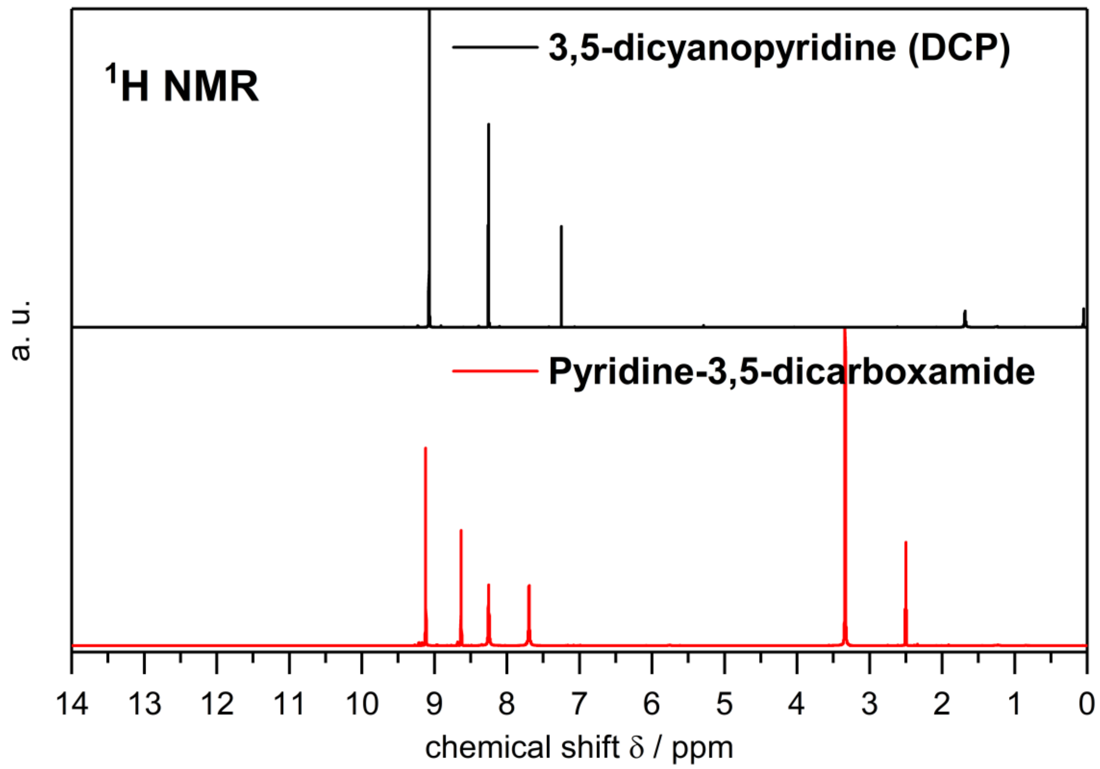

Figure S1 ^{1}H NMR spectra of Pyridine-3,5-dicarboxamide and 3,5-dicyanopyridine.

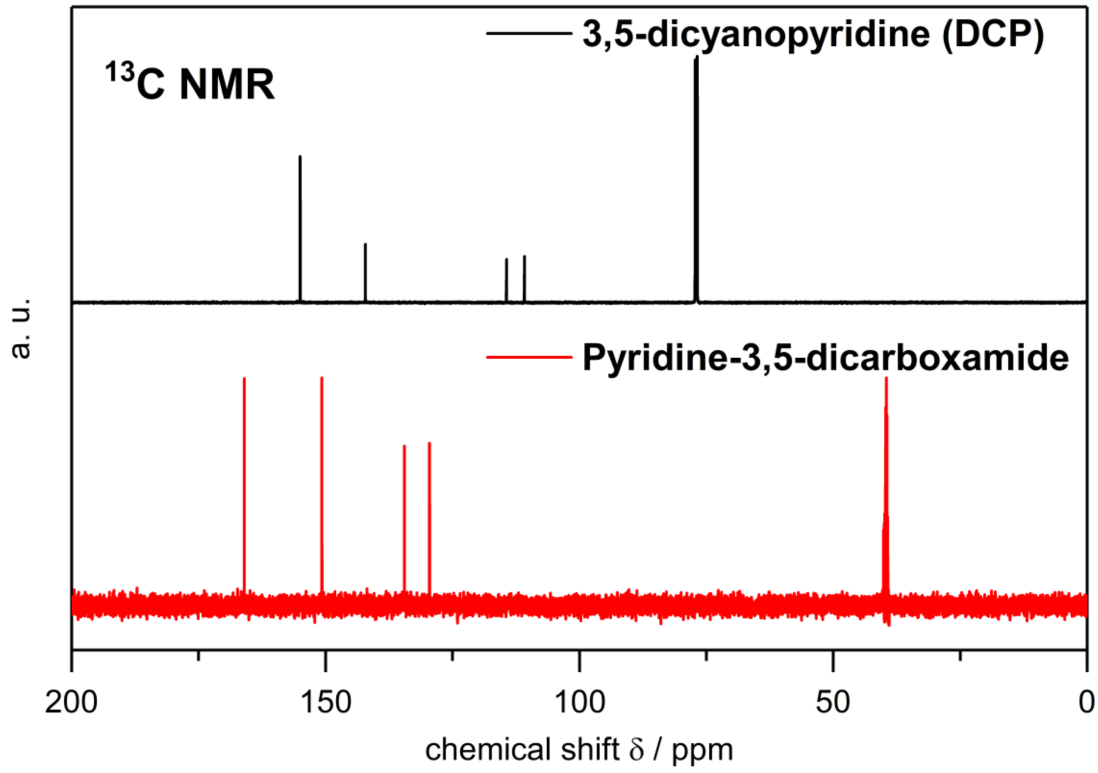

Figure S2 ^{13}C NMR spectra of Pyridine-3,5-dicarboxamide and 3,5-dicyanopyridine.

2. CTF Synthesis

Scheme 1 Temperature protocol of different CTF syntheses.

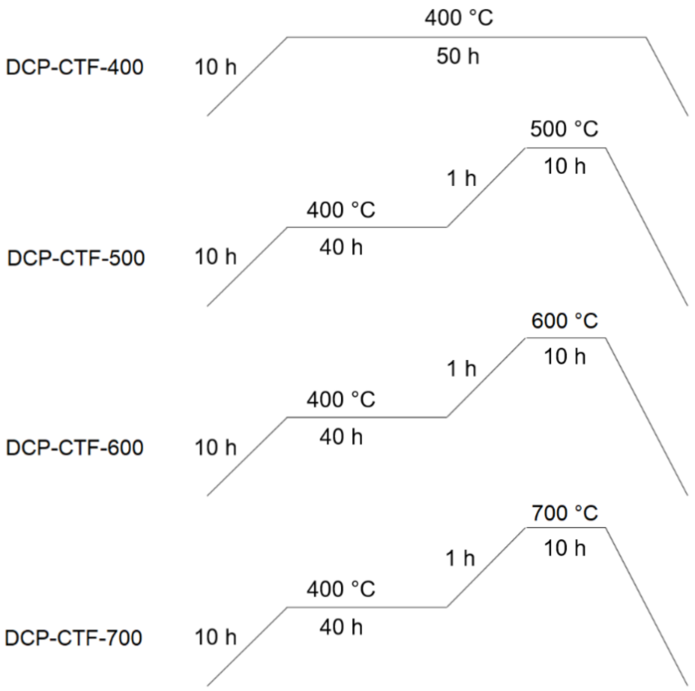

3. Elemental analysis (EA)

Table S1 Elemental analysis of investigated CTF materials.

Sample	Found (%)					Calculated (%)				
	C	H	N	C/H	C/N	C	H	N	C/H	C/N
**ideal**						65.1	2.3	32.5	2.3	2.3
**DCP-CTF-400**	61.6	2.6	28	2.0	2.6	65.1	2.3	32.5	2.3	2.3
**DCP-CTF-500**	57.4	1.5	23.5	3.2	2.9	65.1	2.3	32.5	2.3	2.3
**DCP-CTF-600**	48.9	1.0	15.8	4.1	3.6	65.1	2.3	32.5	2.3	2.3
**DCP-CTF-700**	75.32	0.7	12.7	8.5	6.9	65.1	2.3	32.5	2.3	2.3

4. Argon physisorption

Table S2 Porosity data of the investigated CTF samples including their specific surface area (argon), micropore volume, mesopore volume, ratio of micropore and mesopore volume and total pore volume (at p/p_0 = 0.95).

Sample	Gas	S_{BET} $m^2\ g^{-1}$	V_{Micro} $cm^3\ g^{-1}$	V_{Meso} $cm^3\ g^{-1}$	V_{Micro}/V_{Meso}	V_{Total} $cm^3\ g^{-1}$
**DCP-CTF-400**	Ar	680	0.26	0.03	8.7	0.30
**DCP-CTF-500**	Ar	1120	0.45	0.03	15.0	0.50
**DCP-CTF-600**	Ar	1940	0.80	0.11	7.3	0.93
**DCP-CTF-700**	Ar	3120	1.08	0.54	2	1.66

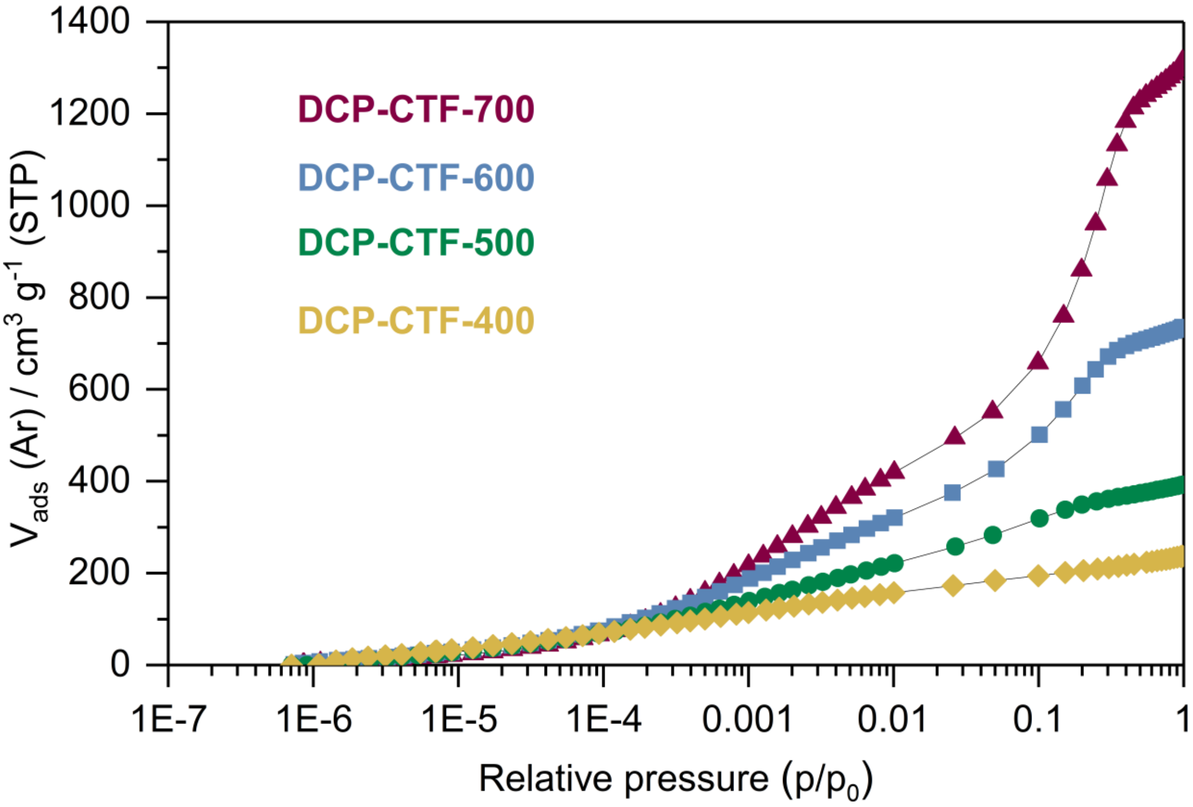

Figure S3 Argon physisorption isotherm (87 K) of DCP-CTF-400, DCP-CTF-500, DCP-CTF-600 and DCP-CTF-700. X-axis is given in logarithmic scale.

5. Nitrogen physisorption (77 K)

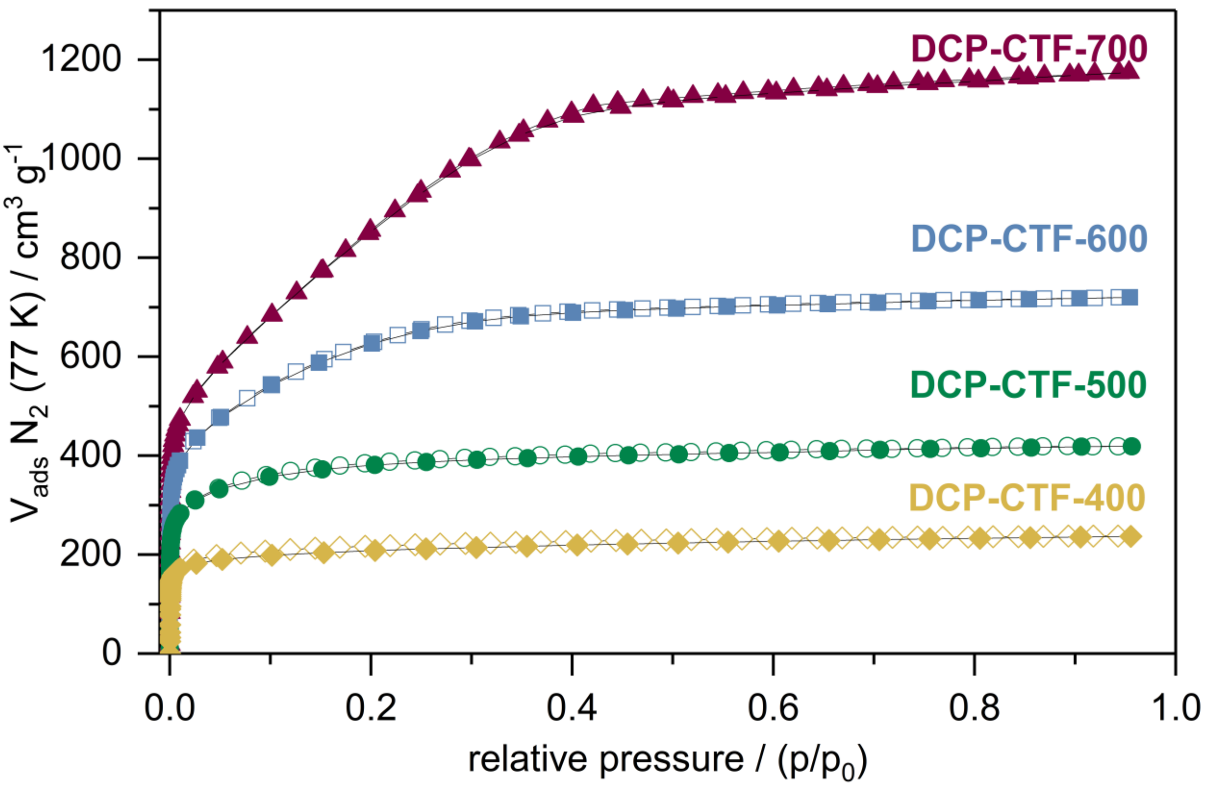

Figure S4 Nitrogen physisorption isotherm (77 K) of DCP-CTF-400, DCP-CTF-500, DCP-CTF-600 and DCP-CTF-700.

6. Pore Size Distribution (N_2)

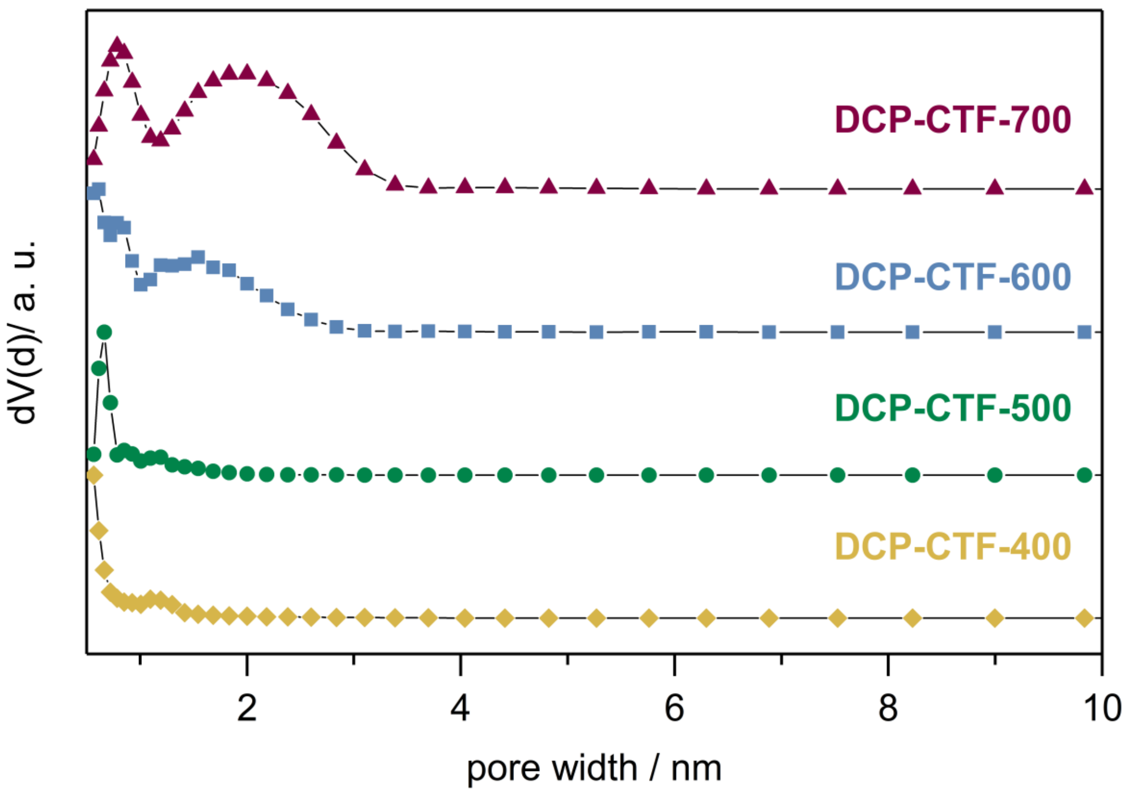

Figure S5 Pore size distribution (QSDFT) derived from nitrogen physisorption (77 K).

7. Carbon dioxide physisorption (273 K)

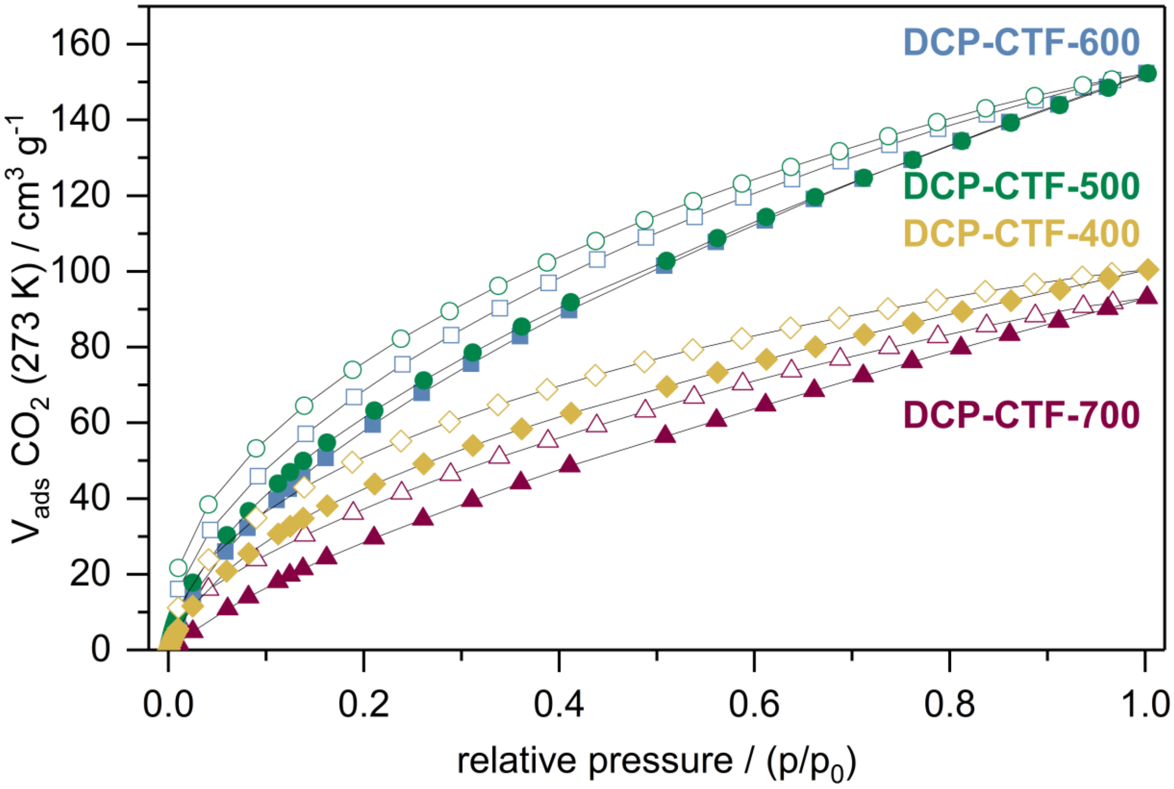

Figure S6 Carbon dioxide physisorption (273 K) of DCP-CTF-400, DCP-CTF-500, DCP-CTF-600 and DCP-CTF-700.

8. X-ray photoelectron spectroscopy

Table S3: Position, FWHM, peak area and procentual peak area derived from C 1s and N 1s deconvolution.

Sample	C 1s				N 1s			
	Posit. / ev	FWHM / ev	Area	%Area	Posit. / ev	FWHM / ev	Area	%Area
DCP-CTF-400	284.20	1.80	2498.36	70.73				
	286.30	1.80	685.17	19.39	398.00	1.64	1485.20	80.96
	288.20	1.80	84.09	2.38	399.53	1.64	349.34	19.04
	285.50	1.80	265.20	7.51				
	284.70	1.80	0.00	0.00				
DCP-CTF-500	284.20	1.62	921.85	28.31				
	286.30	1.62	530.03	16.27	398.00	1.45	883.98	54.59
	287.83	1.62	238.30	7.31	399.47	1.45	459.80	28.38
	285.50	1.62	543.28	16.68	400.48	1.45	275.99	17.03
	284.40	1.62	1023.91	31.44				
DCP-CTF-600	284.20	1.32	507.71	18.54	398.00	1.42	566.70	47.54
	286.30	1.32	420.04	15.33	399.54	1.42	317.67	26.64
	287.72	1.32	199.24	7.27	400.69	1.42	252.49	21.17
	285.50	1.32	508.82	18.58	402.33	1.42	55.61	4.66
	285.03	1.33	1102.83	40.27				
DCP-CTF-700	284.20	1.23	1581.54	37.96	397.86	1.56	455.51	36.44
	286.30	1.23		10.31	399.50	1.56	289.69	23.17
	287.49	1.23	430.02	5.60	400.71	1.56	280.51	22.43
	285.50	1.23	233.49	17.90	402.10	1.56	97.83	7.82
	285.14	1.22	746.11	28.23	403.42	1.56	71.50	5.71

	1176.6<br>9	405.02	1.56	55.49	4.43

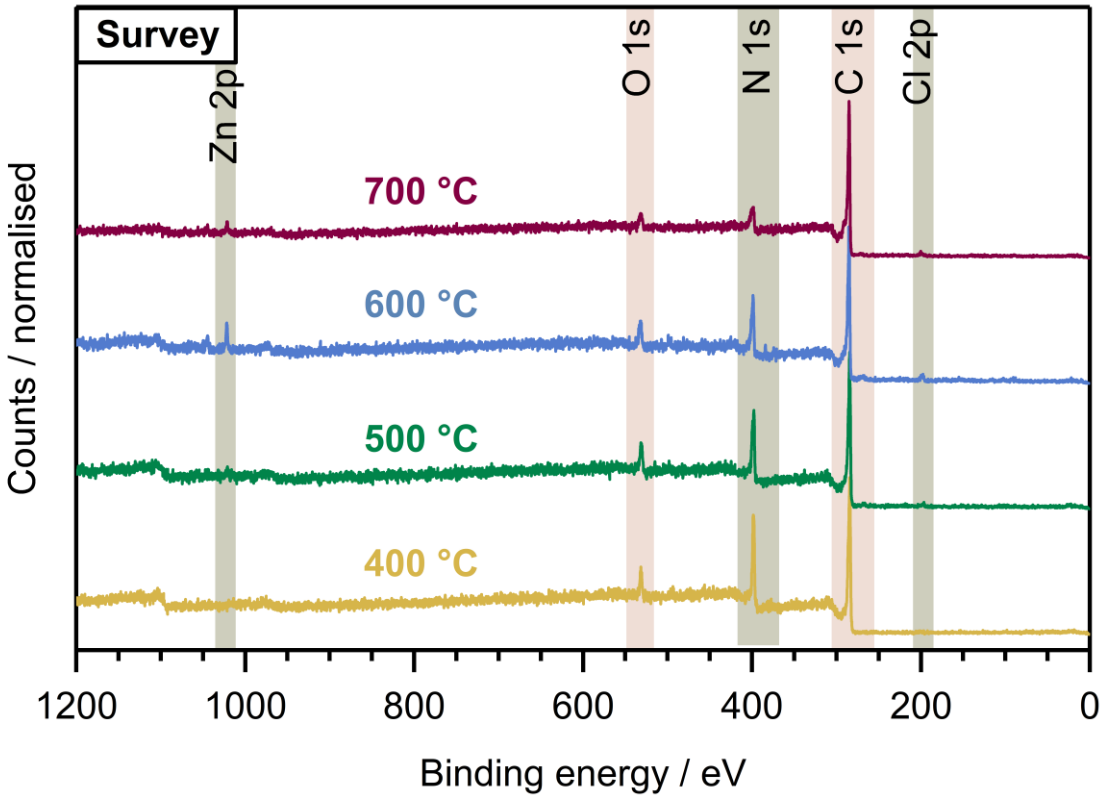

Figure S7 Survey XPS spectra of DCP-CTF-400, DCP-CTF-500, DCP-CTF-600 and DCP-CTF-700.

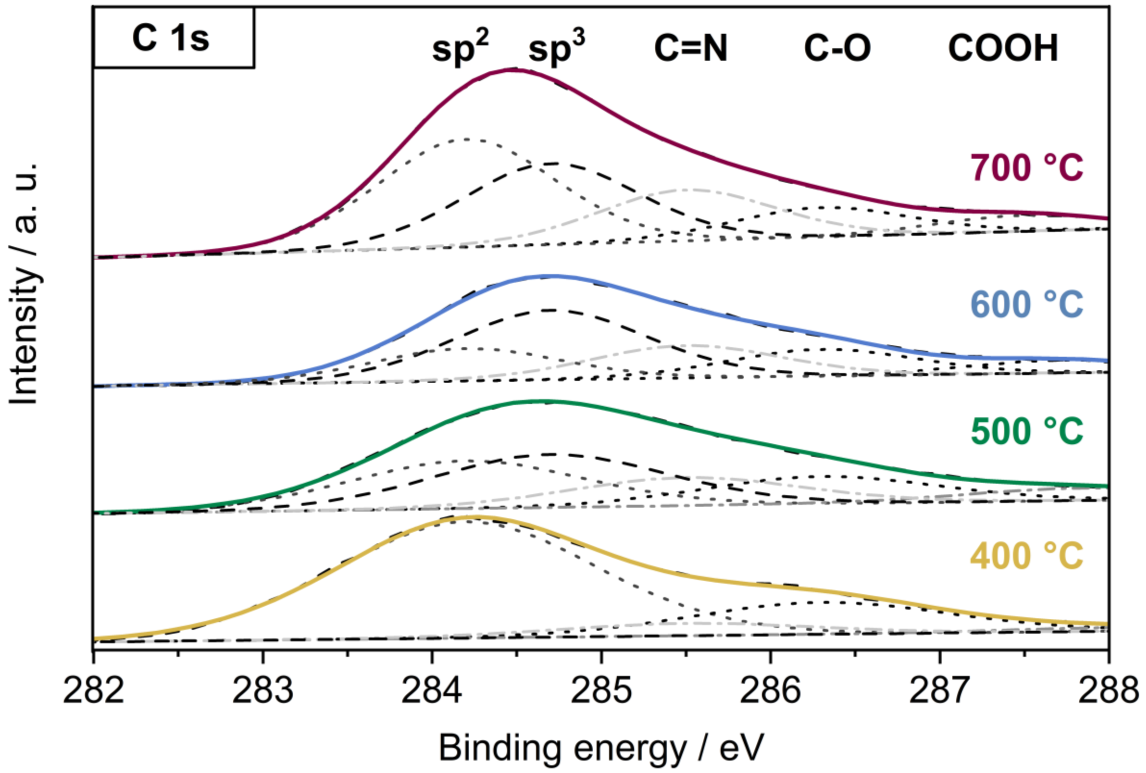

Figure S8 Deconvoluted high resolution C 1s spectra of DCP-CTF-400, DCP-CTF-500, DCP-CTF-600 and DCP-CTF-700.

9. Raman spectroscopy

Table S4: Raman fitting data of DCP-CTF-500, DCP-CTF-600 and DCP-CTF-700.

Sample	Peak height		Area		All area	
**DCP-CTF-500**	I(D1)/I(G1)	1.80	I(D1)/I(G1)	5.54	I(D)/I(G)	3.88
	I(D2)/I(G2)	0.23	I(D2)/I(G2)	2.31		
	I(G2)/I(G1)	0.78	I(G2)/I(G1)	1.06		
**DCP-CTF-600**	I(D1)/I(G1)	1.89	I(D1)/I(G1)	6.48	I(D)/I(G)	2.68
	I(D2)/I(G2)	0.19	I(D2)/I(G2)	0.36		
	I(G2)/I(G1)	0.88	I(G2)/I(G1)	1.63		
**DCP-CTF-700**	I(D1)/I(G1)	1.74	I(D1)/I(G1)	6.76	I(D)/I(G)	2.60
	I(D2)/I(G2)	0.22	I(D2)/I(G2)	0.40		
	I(G2)/I(G1)	0.87	I(G2)/I(G1)	1.89		

10. Infrared spectroscopy (IR)

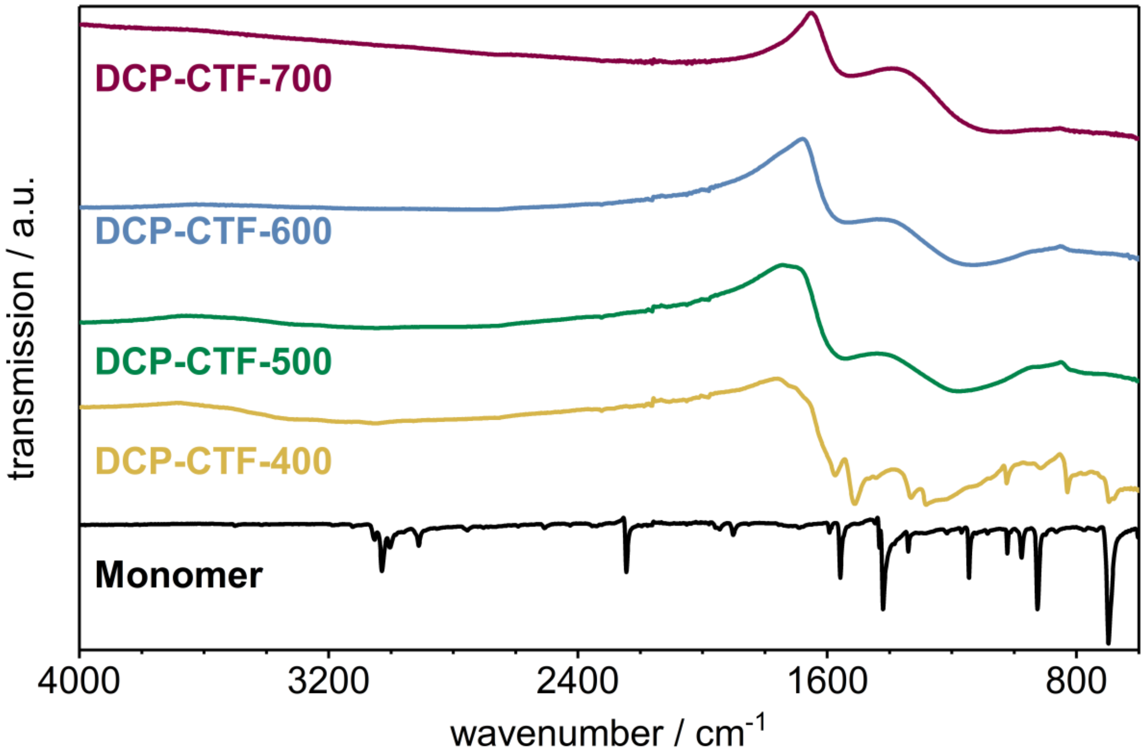

Figure S9 FT-IR spectra of DCP-CTF-400, DCP-CTF-500, DCP-CTF-600 and DCP-CTF-700.

11. Powder X-ray diffraction (PXRD) patterns

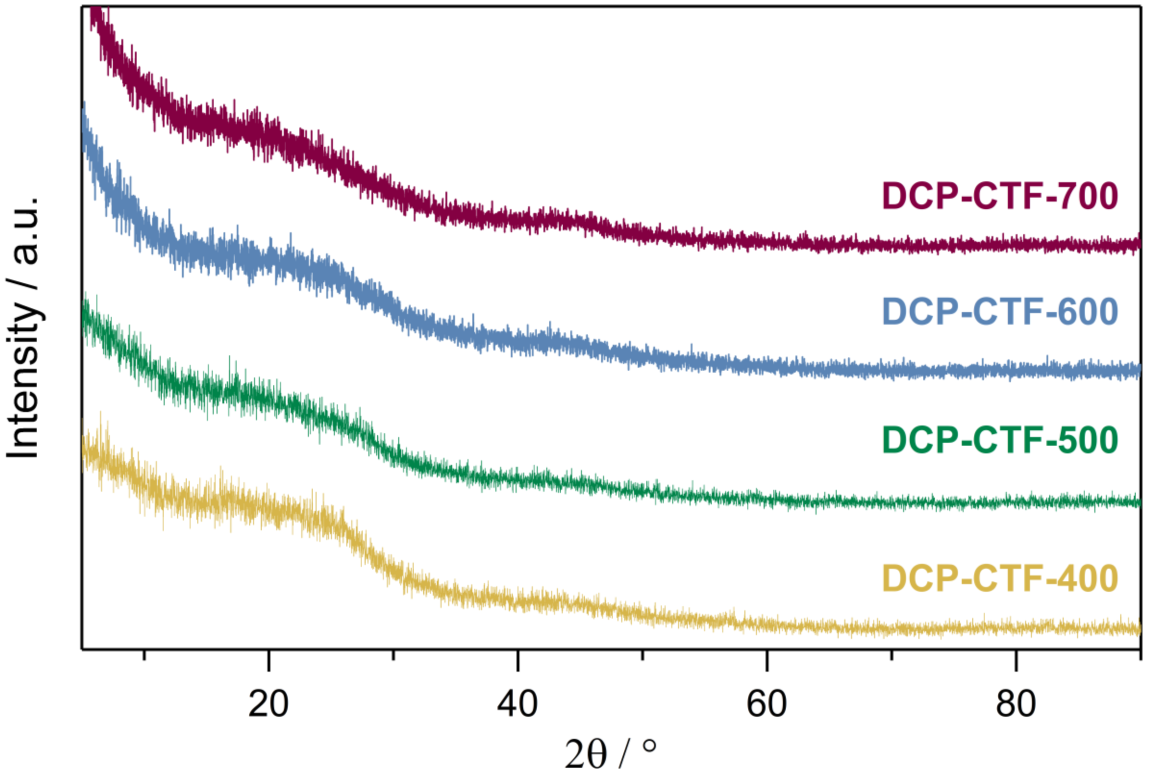

Figure S9 PXRD data of DCP-CTF-400, DCP-CTF-500, DCP-CTF-600 and DCP-CTF-700.

12. Thermogravimetric analysis

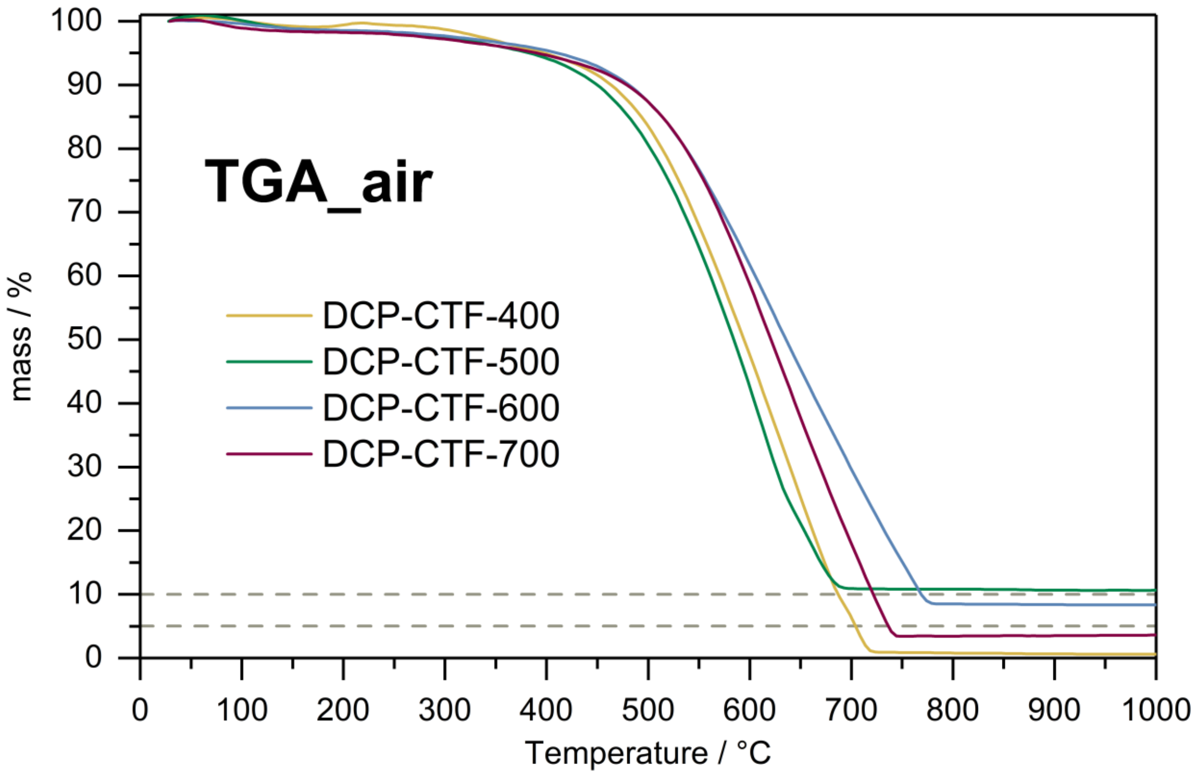

Figure S10 TGA data (air) of DCP-CTF-400, DCP-CTF-500, DCP-CTF-600 and DCP-CTF-700.

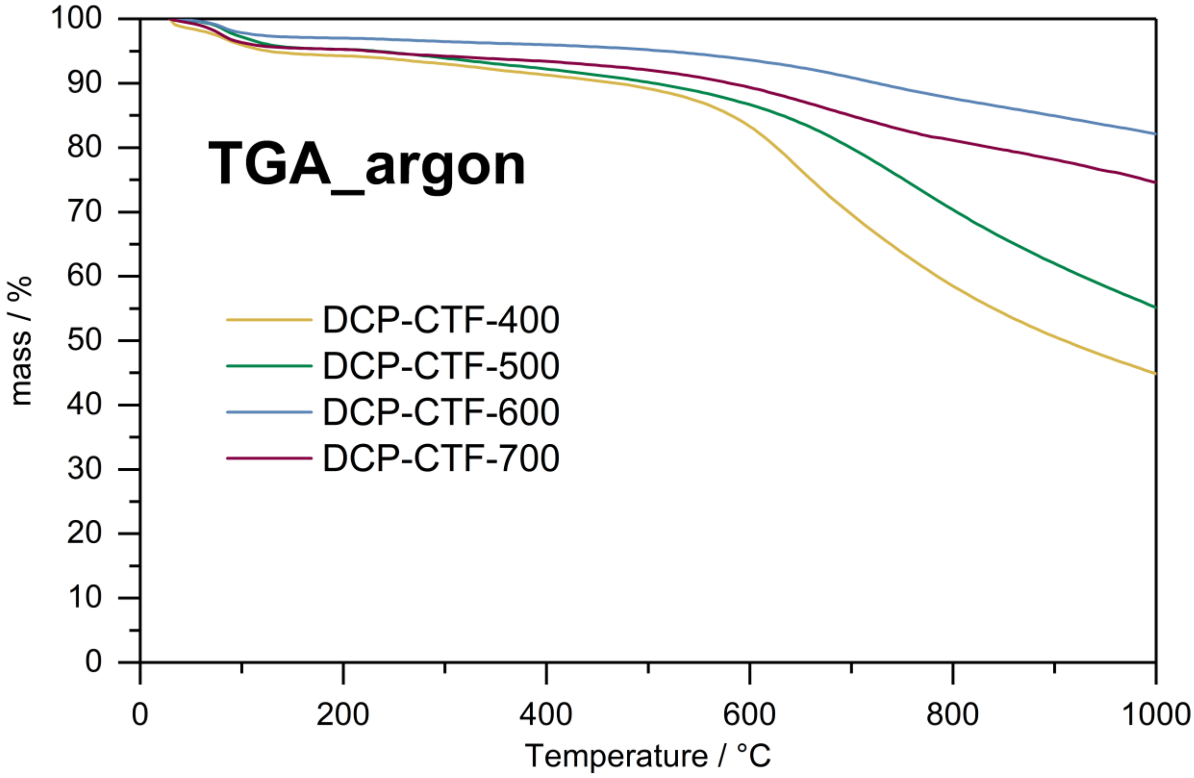

Figure S11 TGA data (argon) of DCP-CTF-400, DCP-CTF-500, DCP-CTF-600 and DCP-CTF-700.

13. Scanning electron microscopy (SEM)

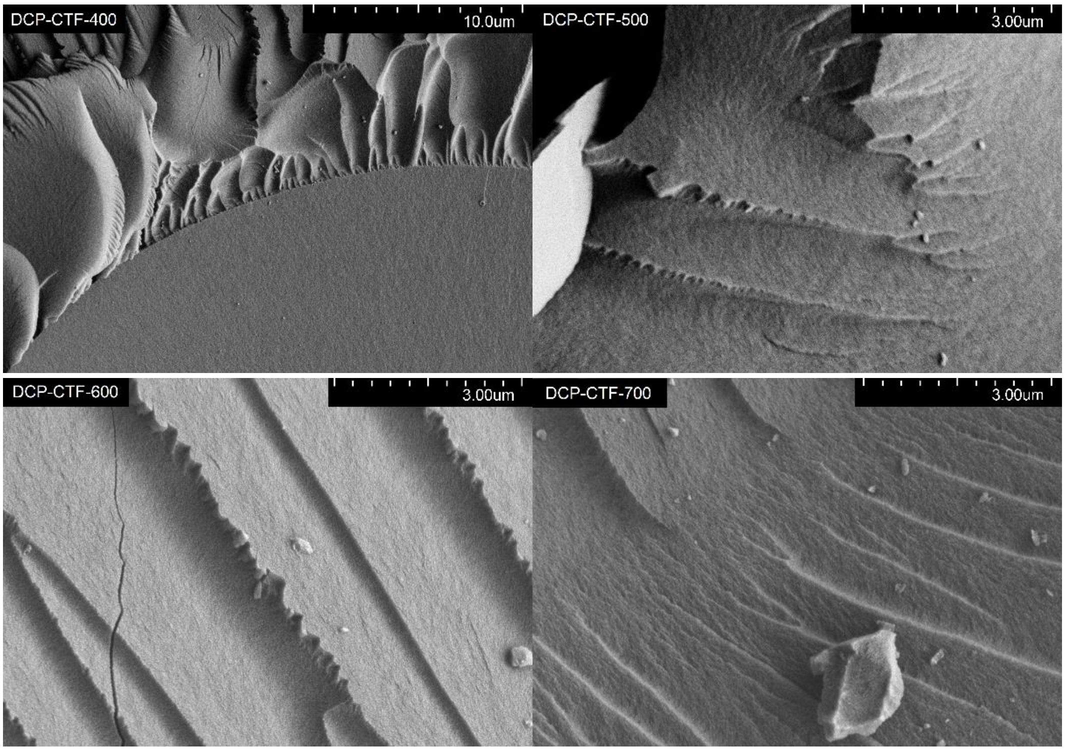

Figure S12 SEM images of DCP-CTF-400, DCP-CTF-500, DCP-CTF-600 and DCP-CTF-700 after washing.

14. Electrochemical characterization

Electrochemical characterization of sample DCP-CTF-700 measured in 1 M Li_2SO_4 electrolyte.

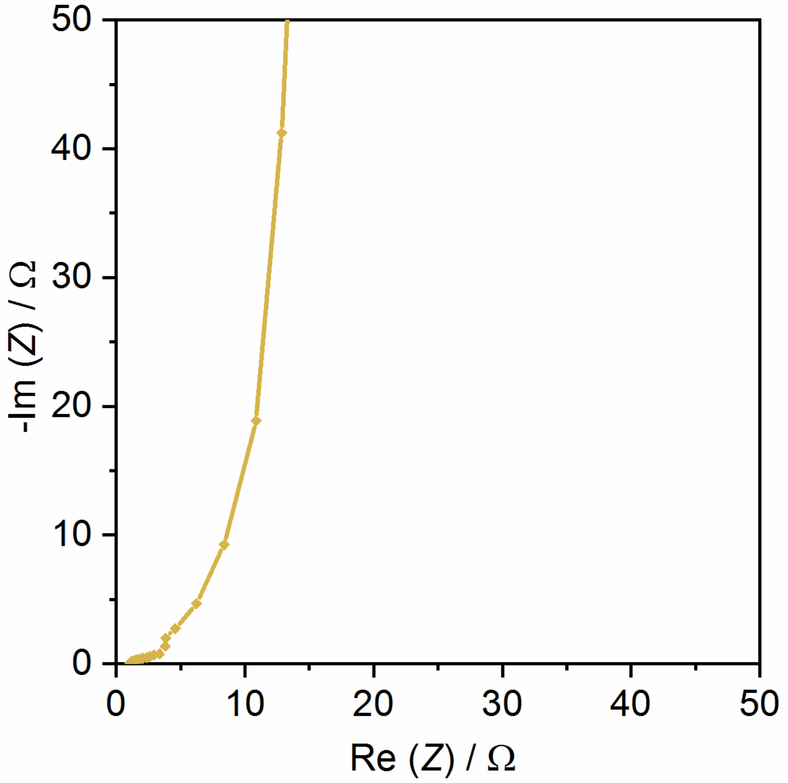

Figure S13 Nyquist plot of sample DCP-CTF-700 measured in 1 M Li_2SO_4.

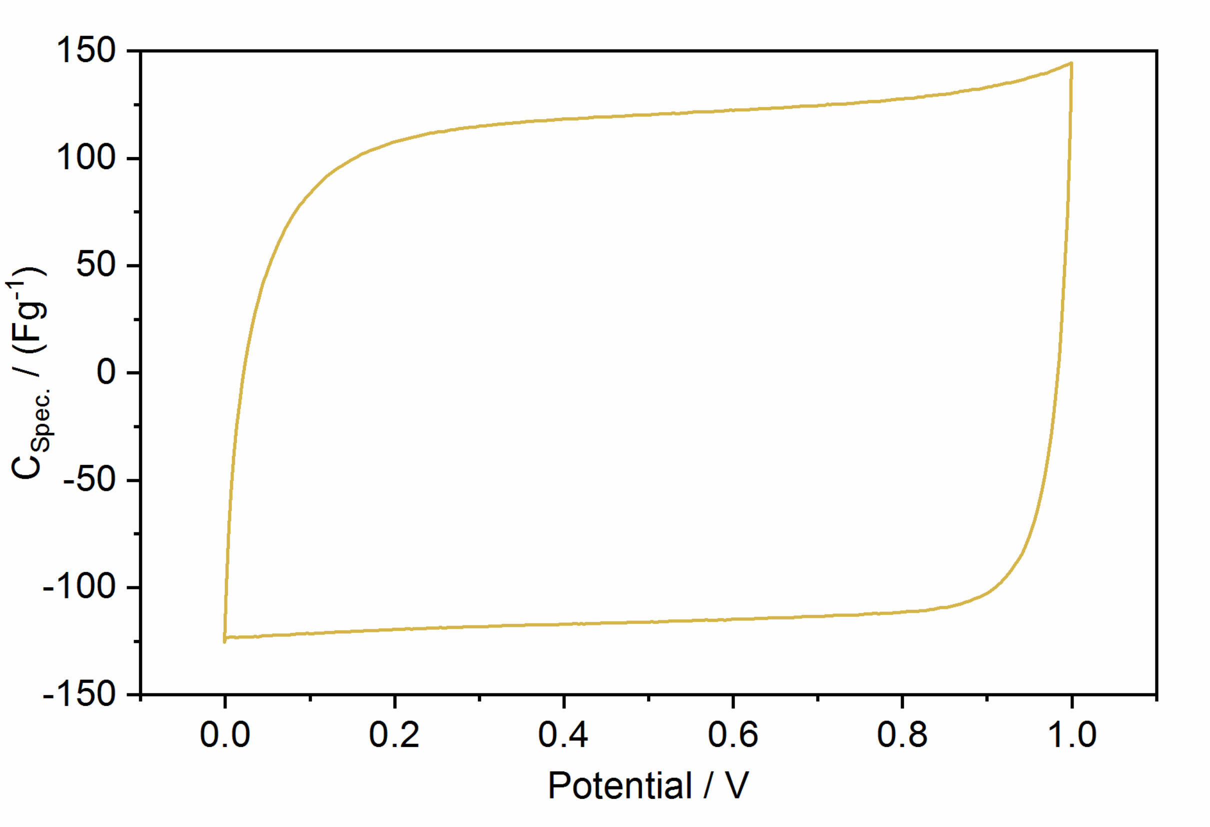

Figure S14 Cyclic voltammogram of sample DCP-CTF-700 measured in 1 M Li_2SO_4. The scan rate is 10 mVs^{-1}.

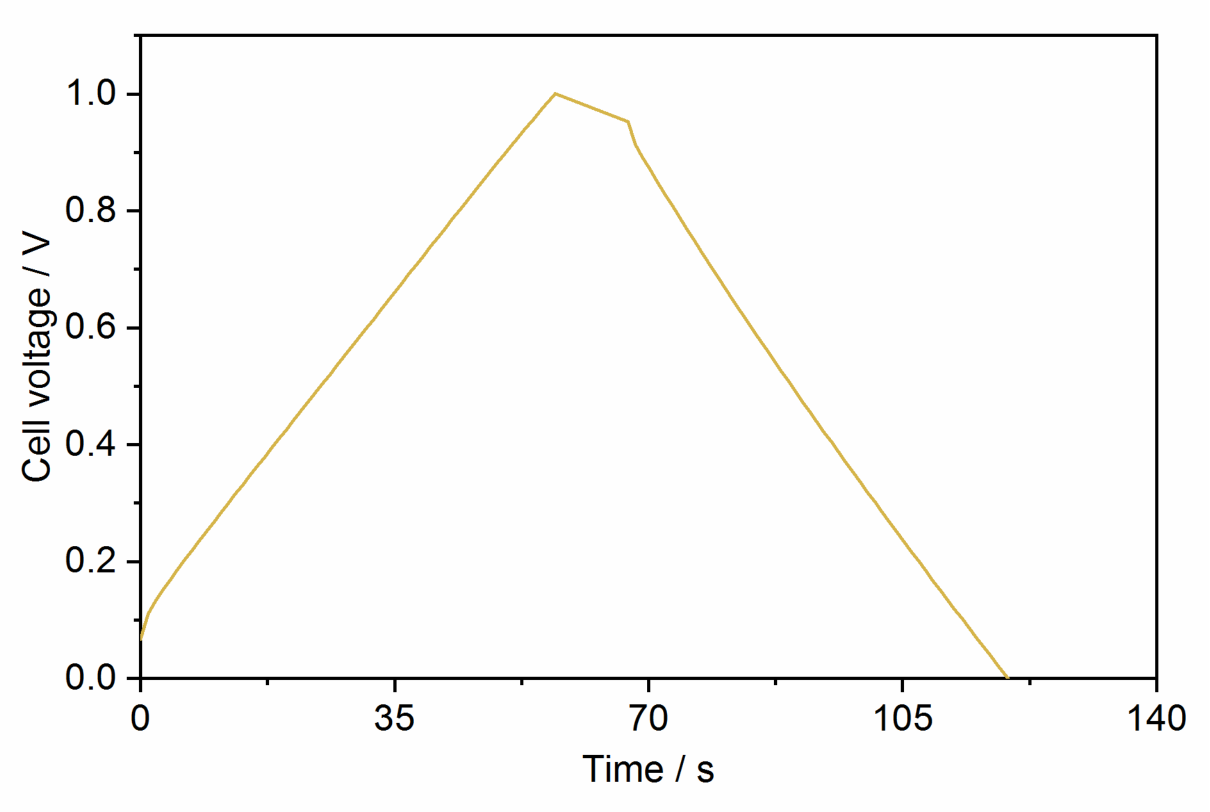

Figure S15 Galvanostatic charge and discharge of sample DCP-CTF-700 measured in 1 M Li_2SO_4. The specific current is 1 Ag^{-1}.

References

1 J. Šturala, S. Boháčová, J. Chudoba, R. Metelková and R. Cibulka, *J. Org. Chem.*, 2015, **80**, 2676–2699.
